# Non-Invasive Brain Stimulation for Perinatal Depressive Disorder: A Literature Review

**DOI:** 10.1192/j.eurpsy.2024.1460

**Published:** 2024-08-27

**Authors:** C. Pinheiro Ramos, M. Alves, D. Durães, S. Mendes, A. Gamito

**Affiliations:** Setúbal Hospital Center, Setúbal, Portugal

## Abstract

**Introduction:**

Peripartum Depressive Disorder (PPD) is a Peripartum Mental Disorder (PMD) characterized as a Major Depressive Disorder (MDD), wherein the manifestation of depressive symptoms initiates either during pregnancy or within the first 12 months following childbirth.

PPD impacts both maternal well-being and infant health, resulting in unfavorable outcomes during pregnancy and the postpartum period.

Non-Invasive Brain Stimulation (NIBS) is one of the rapidly expanding fields in medicine, using a range of techniques to modulate the brain.

**Objectives:**

This study aimed to summarize the latest evidence about the impact of NIBS (efficacy, tolerance, and safety) in PPD.

**Methods:**

A review was conducted, drawing on reputable (PubMed and Web of Science databases).

Key brain stimulation modalities, such as Transcranial Magnetic Stimulation (TMS), Transcranial Electrical Stimulation (TES), and Electroconvulsive therapy (ECT) were analyzed in the context of PPD.

**Results:**

Preliminary findings indicate promising positive effect of NIBS in reducing symptoms associated with PPD.

In the postpartum, the favorable outlook on the effectiveness of NIBS implies that, when feasible, women diagnosed with mild to moderate PPD, especially those reluctant to initiate pharmacological interventions, should be presented with TMS or TES as an alternative therapeutic approach.

However, some doubts persist about the safety of NIBS regarding fetus and preterm birth.

**Conclusions:**

NIBS constitutes a viable option for pharmacological and psychotherapeutic interventions, and it can also be integrated into comprehensive treatment regimens.

Future research include large-scale clinical trials and longitudinal studies is needed to address the efficacy, security, and long-term effects of NIBS.

**Disclosure of Interest:**

None Declared

